# Expert Consensus Recommendations for Clinical Use of the AYON Body Contouring System in Ultrasound-Assisted Liposuction: A Modified Delphi Study With Early Postmarket Observations

**DOI:** 10.1093/asjof/ojag184

**Published:** 2026-09-23

**Authors:** Paul Vanek, Paul G Ruff, Barry E DiBernardo, Charles J Galanis, Michael Kluska, Melinda Lacerna Kimbrell, David Shafer, Johnny Franco

## Abstract

**Background:**

Ultrasound-assisted liposuction (UAL) is widely used in aesthetic body contouring to facilitate adipose tissue fragmentation and aspiration. Advances in device technology have focused on improving energy delivery, procedural efficiency, and workflow integration. The AYON Body Contouring System (Apyx Medical Corporation, Clearwater, FL; AYON UAL) is a recently introduced UAL platform that integrates infiltration, ultrasonic fragmentation, and aspiration within a single system.

**Objectives:**

The aim of this study was to develop device-specific expert consensus recommendations for the clinical use of the AYON UAL platform.

**Methods:**

A structured modified Delphi process was conducted with an expert panel of aesthetic surgeons experienced in UAL and clinical use of the AYON UAL platform. Panelists participated in iterative survey rounds and a moderated consensus meeting to evaluate procedural technique, workflow, and safety considerations. Consensus thresholds were predefined, and statements meeting agreement criteria were incorporated into the final recommendations. A concurrent postmarket clinical follow-up (PMCF) survey was analyzed separately to provide descriptive context regarding early real-world platform use.

**Results:**

Consensus was achieved across key aspects of AYON UAL use. Following refinement and consolidation, 19 consensus statements were unanimously confirmed, with additional consensus findings providing practical context across key domains of device use. The PMCF survey demonstrated broad early clinical adoption across a range of body-contouring procedures and infrequent intraoperative device-related issues.

**Conclusions:**

This modified Delphi consensus provides device-specific recommendations for the clinical use of the AYON UAL platform. Together with descriptive early PMCF findings, these recommendations provide a practical framework to support clinical adoption, procedural standardization, education, and future clinical research.

**Level of Evidence: 5 (Therapeutic):**

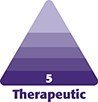

Ultrasound-assisted liposuction (UAL) has been used in aesthetic body contouring for more than 25 years and remains a widely adopted technique for adipose tissue fragmentation and removal.^1-5^ Although the foundational principles of UAL are well established, contemporary platforms incorporate expanded functionality, updated ergonomics, and integrated workflow features that differ from earlier-generation systems.^2,4,6^

Early clinical and experimental investigations established UAL as a technique capable of selectively disrupting adipocytes while largely preserving surrounding connective tissue structures when applied within appropriate exposure parameters.^7-9^ Subsequent studies demonstrated that procedural outcomes depend on factors, including probe motion, depth control, energy delivery, tissue response, and adaptation of treatment parameters to tissue characteristics, anatomic region, and surgical history, reinforcing the importance of surgeon experience and system familiarity.^2,10^

Although the fundamental principles of ultrasound-mediated adipose fragmentation and subsequent aspiration have largely remained consistent across successive UAL device generations, technological refinements have increasingly focused on improvements in probe design, energy modulation, ergonomics, surgical workflow, energy delivery control, and procedural integration.^2,4,11-14^

Historically, UAL procedures often required multiple devices for infiltration, fragmentation, aspiration, and adjunct energy-based treatments. More recent platforms integrate these functions within a single surgical system. The AYON Body Contouring System (Apyx Medical Corporation, Clearwater, FL) is one such platform, incorporating infiltration, ultrasound-assisted fragmentation, and aspiration within a single console while accommodating the Renuvion helium plasma radiofrequency (RF) system (Apyx Medical Corporation) as an integrated adjunctive technology for soft-tissue contraction following liposuction (Figure 1). The ultrasound-assisted functionality of this platform is referred to herein as AYON UAL.

**Figure 1.**
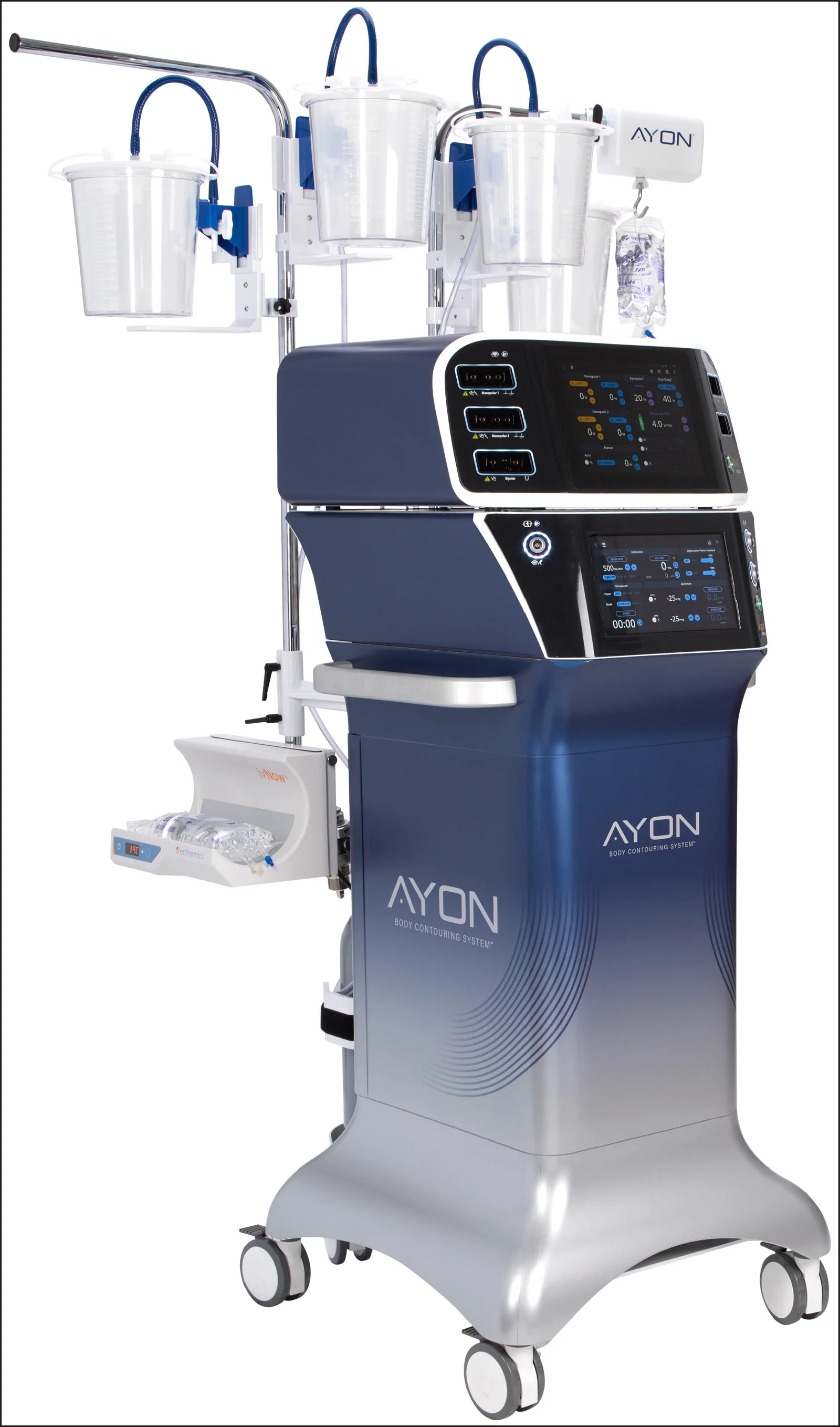
The AYON Body Contouring System: a multimodality surgical platform integrating infiltration, ultrasonic fat fragmentation, and aspiration, with accommodation of the Renuvion system within a single console.

Among its technical features, the AYON UAL platform incorporates integrated workflow functionality together with software-based controls designed to optimize ultrasonic energy, including Liposuction Intelligent Frequency Tracking (LIFT), which adjusts operating frequency in response to probe loading, and a tissue resistance–based feedback mechanism that modulates ultrasonic power output during use. These features may influence procedural workflow and technique but are not specifically addressed in existing consensus recommendations.^11^

There is currently no published clinical guidance addressing AYON UAL–specific best practices or technique considerations. In areas where technological evolution precedes the availability of comparative clinical evidence, structured expert consensus can provide practical guidance for early clinical adoption.^15^ Accordingly, the purpose of this work was to develop expert consensus recommendations for the clinical use of AYON UAL using a structured modified Delphi methodology. A concurrent postmarket clinical follow-up (PMCF) survey provided descriptive context regarding early real-world platform use.

## METHODS

This study employed a structured, modified Delphi methodology to develop expert consensus recommendations for the clinical use of the AYON UAL platform. The process incorporated an initial open-ended exploratory round, structured Likert-scale scoring, and a moderated virtual consensus meeting to iteratively refine and confirm consensus recommendations.^15-17^

The expert panel was comprised of 8 board-certified surgeons with extensive experience in UAL and early clinical experience with the AYON UAL platform. All panelists served as consultants to Apyx Medical and were selected based on their clinical expertise, procedural volume, familiarity with UAL techniques, and willingness to participate in the consensus process. Panelist characteristics, including years in practice, practice setting, liposuction volume, and previous experience with multiple liposuction platforms, were collected during Round 1. Responses were aggregated and de-identified before analysis.

### Question Development and Governance

The Round 1 Delphi questionnaire was developed through an iterative internal process. Initial question domains were informed by previous UAL literature and published consensus recommendations while incorporating AYON UAL–specific topics not addressed in existing guidance.

Draft questions were developed by the project sponsor's clinical affairs, research and development, medical affairs, and regulatory teams and refined through internal review in collaboration with 2 surgeon authors (P.V. and P.G.R.). Revisions focused on clarity, minimizing redundancy, avoiding commercially comparative language, and eliciting technique-focused experiential input. Panelists independently evaluated all statements through anonymous electronic surveys using predefined consensus thresholds. Sponsor personnel synthesized qualitative responses and prepared draft statements for panel review but did not participate in statement scoring or determine consensus outcomes. Statement wording was refined based on aggregated panel feedback and finalized by the panel during the consensus process.

### Delphi Procedure

Delphi Round 1 was conducted in early January 2026 and consisted of 122 open-ended questions distributed electronically using a secure online survey platform (SurveyMonkey, San Mateo, CA). Responses were aggregated and de-identified before analysis. Round 1 responses were synthesized into draft consensus statements organized by procedural domain for quantitative evaluation in Round 2.

Delphi Round 2 was conducted in late January 2026 and consisted of evaluation and scoring of 146 draft consensus statements using a 5-point Likert scale (1 = strongly disagree to 5 = strongly agree). Panelists were instructed to base their responses on typical clinical practice patterns rather than isolated or anecdotal cases and could provide explanatory comments for any statement. Responses were collected electronically, anonymized, and aggregated for analysis.

Consensus thresholds were defined a priori. Agreement was defined as selection of “agree” or “strongly agree” (Likert scores of 4 or 5). Statements achieving agreement by at least 75% of panelists (≥6 of 8) were considered to have reached consensus, whereas agreement by at least 87.5% of panelists (≥7 of 8) was considered high consensus. Statements not meeting these thresholds were reviewed for refinement or exclusion.

Following Round 2, statements meeting the predefined consensus threshold were reviewed, refined, and consolidated based on aggregated panel feedback. Statements representing broadly applicable surgical principles or confirmatory findings were retained as supporting narrative rather than included in the formal consensus statement list, resulting in a streamlined set of proposed consensus statements.

Delphi Round 3 consisted of a moderated virtual consensus meeting held in February 2026 to review the proposed consensus statements derived from Round 2 results and confirm final consensus alignment across all retained statements. Seven of eight panelists participated in the consensus meeting, and the eighth confirmed consensus electronically. Minor wording edits were incorporated without changing the agreed intent of the statements. The final consensus statements were circulated electronically to all panelists for final review. No additional revisions were requested, and unanimous consensus was confirmed.

“Clinical pearls/expert insights” identified during the Delphi process were summarized separately from the formal consensus statements to reflect shared expert judgment.

### Postmarket Clinical Follow-up Survey

A PMCF survey was conducted by Apyx Medical between August 2025 and January 2026 during the early commercialization of the AYON UAL platform in the United States. The survey collected technical, procedural, and day-of-surgery adverse event data from 19 clinical sites, including those of the Delphi panelists. The PMCF data were analyzed descriptively and independently of the Delphi process and were not used to generate, weight, or modify consensus statements.

### Ethical Considerations

This project did not involve research on human participants as defined by applicable regulatory guidance. The Delphi process captured expert opinion only, and the PMCF survey collected nonidentifiable procedural and adverse event data for descriptive analysis. No identifiable patient-level data were collected.

## RESULTS

A total of 8 board-certified plastic surgeons participated in the Delphi consensus process (Table 1). Panelists represented diverse private practice settings and had extensive experience with UAL and multiple liposuction platforms.

**Table 1.** Delphi Panel Characteristics

Characteristic	Summary
Panel size	8 surgeons
Specialty	Plastic surgery (8/8)
Practice setting	Solo (*n* = 4); group (*n* = 3); multisurgeon center (*n* = 1)
Years in practice	13-35 years
Liposuction experience (any modality)	12 to >30 years
UAL experience	3 to >25 years
Previous liposuction platform experience	VASER (multiple generations), PAL, LAL, RF-assisted devices
Annual liposuction volume	∼100-400 cases/year (mean annual liposuction volume of 230 cases/year)
Proportion of cases using UAL	∼30% to nearly 100%
Revision liposuction performed	Yes (reported by most); estimated 5%-25% of cases
High-definition liposculpture	Performed by most panelists (variable proportion)
Reported drivers for AYON adoption	Platform consolidation, ultrasound advancements, workflow efficiency, and integration of body-contouring systems

LAL, laser-assisted liposuction; PAL, power-assisted liposuction; RF, radiofrequency; UAL, ultrasound-assisted liposuction.

### Delphi Consensus Results

Of the 146 statements evaluated in Round 2, 95.9% (140/146) met the predefined consensus threshold (≥75%), including 81.5% (119/146) that achieved high consensus (≥87.5%). Following refinement and consolidation, 19 final consensus statements (Table 2) were unanimously confirmed during the final Delphi round.

**Table 2.** Final Unanimous AYON UAL Consensus Statements

Procedural domain	Statement #	Final consensus statement
Pretreatment/patient selection	1	AYON UAL may be employed in most liposuction cases, including fibrotic and revision cases, with broader application (eg, high-definition liposculpture) expanding as surgeon experience increases.
Infiltration strategy	2	Tumescent solutions may be warmed before infiltration to help maintain adequate body temperature, particularly in high-volume liposuction cases.
Infiltration strategy	3	Adequate and uniform tumescent infiltration extending beyond marked treatment boundaries is critical for safe, efficient, and effective AYON UAL performance, with infiltration volume determined by treatment area, procedural extent, tissue characteristics, and surgeon preference.
Ultrasound technique	4	During AYON UAL, tissue response during fragmentation is assessed using tactile feedback, visual cues, and changes in tissue resistance, with adequate application determined by loss of resistance, improved tissue compliance, and ease of probe passage.
Ultrasound technique	5	AYON UAL technique is adapted based on anatomic region and tissue characteristics, with increasing experience improving endpoint recognition and procedural efficiency.
Probe selection	6	Probe selection during AYON UAL is tailored to anatomic region and access considerations; generally, smaller-diameter probes are favored for face and neck applications, whereas larger-diameter probes are preferred for trunk, extremity, and higher-volume regions.
Probe selection	7	Higher ring count probes are favored for broader, less fibrotic regions, whereas fewer rings tend to be used in dense or fibrotic tissue.
Suction/aspiration	8	Completion of aspiration during AYON UAL is guided by intraoperative assessment of aspirate characteristics (eg, quality of effluent, bleeding) and real-time endpoint evaluation using tactile and visual cues (eg, pinch test and cannula glide), in conjunction with contouring goals.
Fat handling and aspirate management	9	Aspirate obtained during AYON UAL procedures is handled using standard fat processing practices consistent with existing liposuction workflows and established practice patterns.
Integration with adjunctive energy-based devices	10	AYON UAL may be combined with adjunctive energy-based devices when clinically indicated based on patient characteristics and aesthetic goals.
Integration with adjunctive energy-based devices	11	When adjunctive energy-based device treatment is planned following AYON UAL, safety considerations include confirmation of adequate tissue hydration, monitoring tissue response, allowing appropriate thermal recovery between energy applications, and maintaining awareness of total cumulative energy delivery across devices to minimize complication risk (eg, seroma formation, dermal or subcutaneous fibrosis).
Settings, modes, and power selection	12	Standard mode is used as the default AYON UAL mode, whereas continuous mode may be used for select situations such as dense or fibrotic tissue, revision cases, high BMI patients, or large-volume regions.
Settings, modes, and power selection	13	Mode, power, and treatment duration are adjusted intraoperatively based on tactile feedback, tissue resistance, and probe depth.
Workflow sequencing	14	The AYON platform streamlines OR workflow by consolidating multiple procedural functions into a single platform. Attributes include standardized tubing, ports, cannulas, disposables, and back-table organization, thereby reducing setup complexity and OR clutter.
Procedural safety considerations	15	Skin-port protectors are used during ultrasound application and aspiration when cannula size allows. Ultrasound activation is avoided during probe insertion or withdrawal through the port to reduce the risk of entry-site thermal or mechanical injury.
Procedural safety considerations	16	AYON UAL application is performed using controlled, methodical probe motion and incremental layering by tissue plane. This technique is modified in thinner tissue treatment areas through reduced energy delivery, limited superficial passes, and heightened awareness of probe depth.
Anatomic-specific considerations	17	AYON UAL technique is adapted across the body based on tissue thickness, density, fibrosis, tissue planes, and proximity to critical structures.
Revision cases/fibrous tissue	18	Revision liposuction cases and fibrotic tissues require a more deliberate, staged AYON UAL approach, often requiring increased treatment time rather than escalation of energy.
Postoperative considerations	19	AYON UAL does not alter standard postoperative care compared with other liposuction techniques.

OR, operating room; UAL, ultrasound-assisted liposuction.

### Additional Consensus Findings

Additional consensus findings are summarized below to provide context for the final consensus statements and common expert practice patterns.

### Pretreatment/Patient Selection

AYON UAL does not require specialized pretreatment beyond routine preoperative evaluation used for standard liposuction procedures. Preoperative counseling and expectation-setting were identified as essential components of patient preparation.

Tissue characteristics, including fat density, fibrosis, and pinch test findings, were considered the primary determinants of candidacy and procedural planning. BMI, skin quality, and tissue laxity also influenced treatment planning, particularly when assessing expected skin contraction and the potential need for adjunctive or excisional procedures; elevated BMI alone was not viewed as a contraindication. Anesthesia selection was guided by treatment extent, number of areas treated, medication considerations, and overall patient safety. Patients with significant chronic edema, lymphedema, or severe venous disease were generally considered suboptimal candidates.

### Infiltration Strategy

Standard tumescent or super-wet infiltration was consistently performed as the initial procedural step when using AYON UAL. Surgeons emphasized safety safeguards, including weight-based calculation of maximum allowable lidocaine dose before infiltration, confirmation during procedural time-out, and ongoing monitoring with adjustment as needed based on procedural duration and extent. Use of tranexamic acid, administered intravenously, within the tumescent solution, or both, was also supported based on patient-specific risk factors and surgeon preference. AYON's integrated infiltration capability was also viewed as improving procedural efficiency through streamlined setup, volume tracking, and workflow continuity.

Infiltration strategies were individualized based on anatomic region, tissue characteristics, and treatment history, including revision and fibrotic cases. Multiplanar infiltration was commonly used to promote uniform tissue preparation before ultrasound-assisted fragmentation. Adequate vasoconstriction was achieved by staging treatment areas rather than relying on a fixed dwell period. In thinner or more delicate regions (eg, face and neck), infiltration volumes were more conservative. Adequacy of infiltration was assessed using tissue turgor and visual blanching, together with typical infiltration-to-aspirate ratios of ∼1.5:1 to 2:1. Tumescent composition varied based on anesthesia type and anticipated procedural duration.

### Ultrasound Technique

Ultrasound energy in AYON UAL is used to facilitate gentle adipose tissue fragmentation to support subsequent aspiration and is incorporated into standard liposuction workflows without requiring a fundamentally different procedural approach. Although tactile endpoints remained the primary determinant of adequate fragmentation, time-based heuristics (eg, treatment time per area or infiltrated volume) were also used to support procedural consistency. Surgeons emphasized the use of multiple access points and varied vectors to promote uniform treatment and reduce the risk of parallel tracking. The nondominant (guide) hand was considered essential for monitoring probe depth, tissue thickness, and skin temperature, preventing end hits, and providing compression to facilitate controlled energy delivery.

Common technical errors included overtreatment beyond the endpoint, inadequate tissue wetting, insufficient port protection, probe dwell in superficial planes, and lapses in attention while the pedal is engaged. Ultrasound power settings were adjusted based on anatomic region and tissue characteristics, with lower settings commonly applied in thinner or more delicate regions such as the face and neck and higher settings used in thicker or more fibrotic tissue where greater resistance is anticipated.

### Probe Selection

Probe selection was strongly influenced by tissue characteristics, particularly fat density, fibrosis, and treatment history, including revision cases where tissue planes may be altered and probe excursion limited. Increased resistance to probe passage and fragmentation was identified as a key driver of probe configuration selection. Probe ring count also influenced ultrasound effect and was selected according to tissue resistance and anticipated fragmentation requirements.

Surgeons emphasized the importance of ongoing assessment of probe integrity throughout use. Probe condition was evaluated visually and functionally, including inspection for tip wear, pitting, or smoothing of ring features. Loss of expected tissue response or delayed loss of resistance prompted inspection and replacement when warranted. Contemporary titanium probes used in current-generation UAL systems were considered durable and not prone to frequent wear; when present, wear is typically identifiable by pitting at the probe tip. Surgeons favored replacing worn probes rather than escalating power settings or prolonging treatment time. Increasing experience also enabled more refined probe selection based on tactile feedback, tissue response, and probe behavior across anatomic regions.

### Suction/Aspiration

Aspiration was performed following ultrasound-assisted adipose tissue fragmentation, with the transition to suction guided by intraoperative tissue response, changes in resistance, and surgeon-defined procedural endpoints. Aspiration technique was described as controlled and deliberate to promote uniform fat removal and minimize contour irregularities. Approaches were adapted based on anatomic region, tissue thickness, and case complexity, with additional modification in revision or fibrotic cases to account for altered tissue planes and increased resistance. Cannula selection was tailored to procedural goals and anatomy, and small volumes of adipose tissue could be intentionally preserved to maintain contour and avoid concavities, particularly during high-definition sculpting applications.

Intraoperative cues such as bloody aspirate, increased resistance, or unexpected tissue response prompted reassessment of suction aggressiveness and could warrant limiting aspiration in the affected area. Mercedes-type cannulas were commonly used for body liposuction following AYON UAL, whereas basket, curved, or specialty cannulas were selectively employed for equalization, contour refinement, or anatomically challenging regions. Some surgeons also described advanced cannula designs intended to control negative pressure distribution at the tip/ports to improve fat extraction efficiency while reducing unintended tissue trauma.

### Fat Handling and Aspirate Management

Aspirate management during AYON UAL was adapted based on procedural goals, particularly whether the case involved body contouring alone or planned autologous fat transfer. When fat grafting was anticipated, harvested aspirate was managed according to each surgeon's established fat grafting protocols. AYON UAL was commonly used for fat harvest in moderate- to large-volume fat transfer, including breast and gluteal grafting, with larger body regions typically selected as donor sites. In contrast, AYON UAL was used less frequently for very small-volume harvest (eg, limited facial grafting), where manual techniques were often preferred.

AYON UAL was also considered advantageous for fat harvest in fibrous or previously treated donor areas, where ultrasound-assisted fragmentation could facilitate smoother aspiration. When fat transfer was planned, donor zones were typically pretreated with ultrasound before aspiration. Harvested aspirate was most often managed using closed or predominantly closed systems and processed by gravity decanting or system-specific techniques before reinjection. When fat transfer was not planned, aspirate was disposed of using standard medical waste protocols.

### Integration With Adjunctive Energy-Based Devices

Adjunctive energy-based devices (EBDs) were frequently incorporated following AYON UAL to address skin laxity after fat reduction, particularly in patients with reduced elasticity or clinically apparent laxity. Adjunctive EBDs were also commonly used in patients with minimal baseline laxity as a preventive approach intended to support longer-term skin quality. Common treatment areas included the arms, abdomen, back, medial thighs, calves, neck, flanks, male chest, axilla, mons, and labia majora.

When adjunctive EBD treatment was planned, AYON UAL technique was generally not modified, with emphasis placed on achieving uniform ultrasound treatment and aspiration before energy delivery. Nonbulk heating technologies (eg, helium plasma RF) were used more frequently, and integration of adjunctive EBDs required deliberate case planning to optimize workflow efficiency and align with anesthesia constraints. In cases involving excisional flaps, non-bulk-heating EBD treatment was commonly limited to selected regions or transition zones, with technique adjusted to account for flap perfusion and postoperative healing.

### Settings, Modes, and Power Selection

Ultrasound power selection was tailored to tissue thickness and density, with higher-resistance tissue (eg, fibrotic fat, revision planes, or dense adipose) generally requiring higher power and/or longer treatment duration, whereas softer adipose tissue was typically treated using lower to moderate settings. Surgeons emphasized that settings selection became more refined with increasing AYON UAL experience as recognition of tissue response and procedural endpoints improved.

More conservative power settings and shorter ultrasound exposure were used in anatomically delicate regions such as the face and neck, as well as areas requiring fine contour control (eg, triceps, biceps, and calves), to minimize the risk of overtreatment. When adipose tissue harvest was planned, lower power settings combined with longer exposure times were preferred to facilitate smoother aspiration and support subsequent fat processing, particularly in fibrotic donor regions.

### Workflow Sequencing Considerations

AYON UAL was typically performed using a structured procedural sequence consisting of infiltration, ultrasound-assisted fragmentation, aspiration, and adjunctive energy-based treatment and/or fat handling when clinically indicated. Surgeons emphasized that procedural efficiency and safety depended on coordinated intraoperative workflow, including clear communication with OR staff and consistent tracking of key procedural parameters such as infiltrated volume, ultrasound activation time, power settings, and aspirated volume.

Advance OR planning, including tower positioning, tubing management, and patient positioning, was considered important for supporting efficient multiarea access and minimizing intraoperative repositioning. Staff familiarity with system priming, controls, and mode transitions was viewed as critical for maintaining workflow continuity. Port placement was guided by anatomic access and scar concealment, with routine use of port protectors considered an essential safety practice.

### Procedural Safety Considerations

Safe AYON UAL performance required structured procedural planning and continuous intraoperative vigilance, including monitoring during prolonged procedures with attention to positioning, pressure points, eye protection, urine output, and physiologic parameters. Ultrasound application was guided by real-time tactile feedback, with loss of tissue resistance and improved probe excursion serving as the primary endpoints. Ultrasound was typically discontinued once adequate tissue response was achieved rather than according to fixed time-based formulas, with tactile findings guiding adjustments to mode, power, treatment duration, and probe depth. Use of multiple access ports and varied treatment vectors was also considered important for promoting uniform treatment and reducing the risk of parallel tracking.

Standardized device-related safety practices included pairing smaller-diameter probes with lower power settings in delicate or superficial treatment zones to reduce excessive energy delivery and tissue injury. Routine inspection of probe tips and handpieces for wear or damage, verification of proper probe securement, and reassessment of system setup in response to unexpected changes in tactile feedback, sound, or device behavior were consistently emphasized. Notably, based on the collective early clinical experience of the expert panel, no probe replacements because of wear or damage had been required between the commercial introduction of the AYON UAL platform and the Delphi consensus meeting, although routine inspection remained standard practice. Because AYON UAL operates relatively quietly, deliberate confirmation of activation status and disciplined foot–pedal control were considered important to prevent unintended energy delivery. Cautious superficial treatment progression and conservative energy application were also emphasized in patients with compromised tissue quality, including those with previous radiation exposure or connective tissue disorders.

### Anatomic-Specific Considerations

Thicker or more fibrotic tissue generally tolerated higher energy application and/or longer treatment duration, whereas thinner or more delicate tissue required reduced energy delivery and heightened vigilance. In the face and neck, lower power settings, smaller-diameter probes, and limited superficial passes were used, with ultrasound primarily confined to deeper or subsuperficial planes. Treatment was avoided or applied selectively in patients with extremely thin tissue, minimal adipose volume, or compromised skin quality. Breast-related procedures also required careful preservation of tissue integrity, particularly when excision or flap elevation was planned. In contrast, the back and posterior trunk generally tolerated higher ultrasound energy because of greater tissue thickness and fibrosis, although aspiration was often more conservative to limit bleeding.

The arms required conservative energy application, careful depth control, and avoidance of overtreatment, particularly in distal or thin-skin regions. Treatment of the thighs and calves emphasized contour preservation, avoidance of over-resection, and protection of transition zones. AYON UAL use in the gluteal region was selective, focusing on contour refinement, transition zones, or fat harvest rather than aggressive volume reduction. Additional caution was recommended in regions with thin skin, limited adipose volume, increased edema, or proximity to critical structures, including the neck, inner thighs, calves, mons, and gynecomastia treatment zones, where ultrasound energy was often minimized or avoided in favor of tissue redistribution, minimal aspiration, or alternative techniques. The abdomen and flanks were commonly treated using a deep-to-superficial sequencing strategy, with ultrasound and aspiration restricted to deep or transition zones and avoided in planned surgical flaps or areas of flap undermining to preserve perfusion and wound healing. AYON UAL was avoided in radiated tissue, compromised flaps, or regions with impaired vascularity because of increased risk of delayed healing or tissue injury.

### Revision Cases/Fibrotic Tissue

Previously operated or fibrotic regions required modification of technique to accommodate altered tissue planes and increased resistance. Surgeons emphasized initiating treatment conservatively, commonly beginning with lower power settings, fewer probe rings, or smaller-diameter probes, and escalating only as tissue response permitted. In heavily scarred regions, AYON UAL was often used selectively in combination with adjunctive mechanical techniques (eg, pretunneling, needle subcision, or basket cannula release) to improve probe excursion and facilitate more uniform energy transmission.

Intraoperative cues—including resistance to probe movement, restricted cannula excursion, and transmission of probe motion to the skin surface—guided treatment intensity and sequencing in revision cases. Loss of resistance, improved tissue mobility, and safe probe passage were considered appropriate treatment endpoints. Slower probe motion, wider spacing of passes, and multivector access were commonly used in fibrotic regions to reduce thermal accumulation and minimize focal tissue injury. In revision cases with contour depressions or irregularities, AYON UAL could also be used to prepare surrounding tissue planes before fat grafting rather than aggressively removing additional tissue.

### Postoperative Considerations

Postoperative management following AYON UAL was primarily determined by procedural extent, anatomic regions treated, and whether concomitant excisional, flap-based, or fat grafting procedures were performed. Routine postoperative assessment included evaluation for edema, bruising, contour irregularities, skin quality changes, and fluid collections. Compression garments were routinely used, with duration individualized according to procedure, aspirate volume, and treatment region (often 6-8 weeks following extensive UAL-only procedures); modification, delay, or limitation was recommended following flap-based or fat grafting procedures to avoid compromising tissue perfusion.

Early postoperative follow-up (typically within 1-3 days) was considered valuable for identifying and managing fluid-related complications before they become persistent. Manual lymphatic drainage was commonly recommended to support edema resolution and tissue remodeling, with timing individualized based on treatment extent, drain presence, flap viability, and patient tolerance. When seromas occurred, initial management consisted of clinical evaluation and ultrasound-guided aspiration, with drain placement reserved for recurrent or clinically significant fluid collections. Overall postoperative recovery was generally considered similar to, or faster than, other liposuction platforms with respect to bruising, swelling, and return to baseline activity.

### Clinical Pearls/Expert Insights

Panelists also demonstrated unanimous agreement on several clinical pearls and expert insights derived from practical experience with AYON UAL (Figure 2).

**Figure 2.**
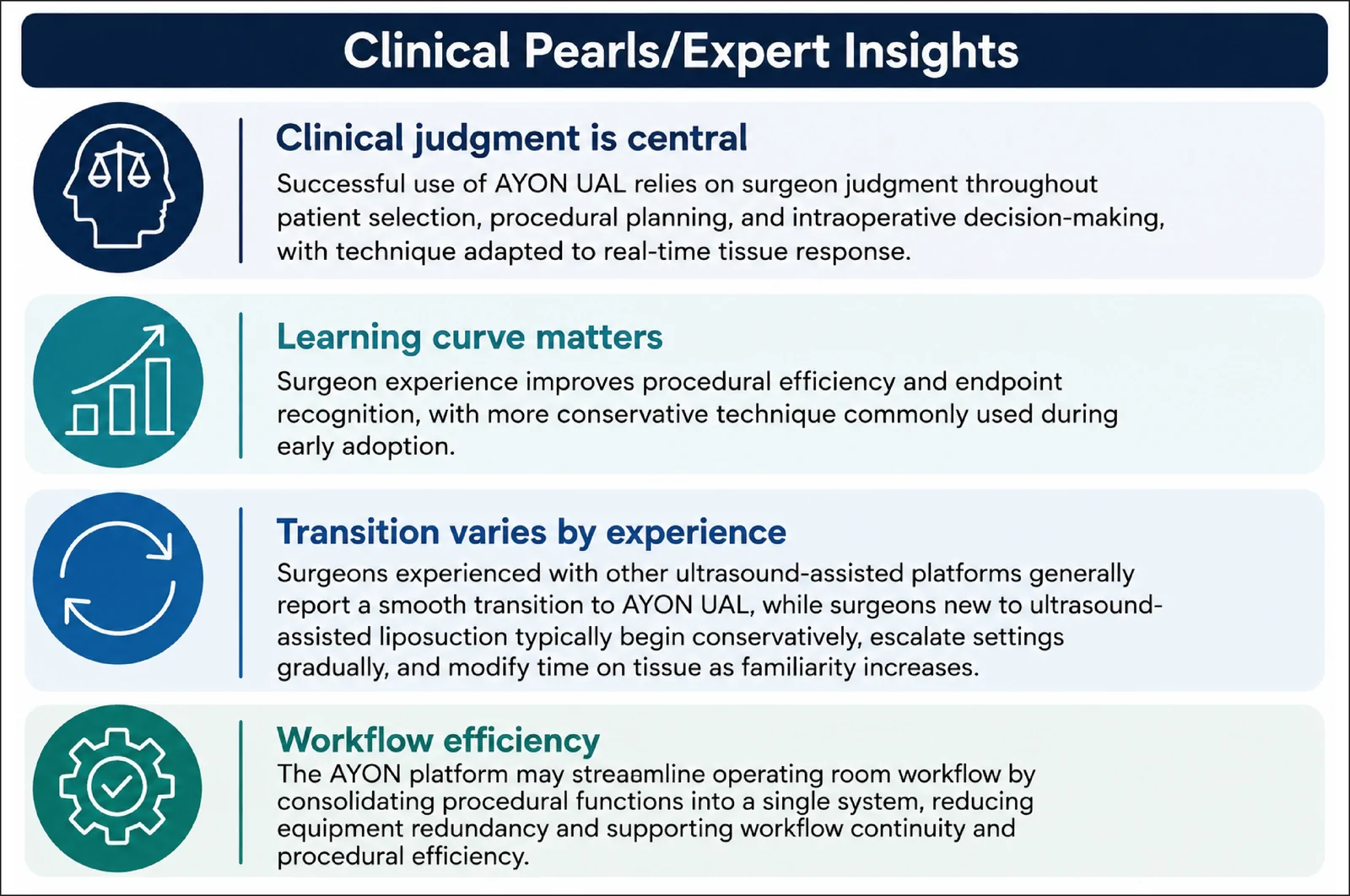
Summary of key practical insights identified by the expert panel during the modified Delphi consensus process. These observations reflect shared clinical experience and judgment regarding AYON UAL adoption, procedural technique, and workflow and are presented as complementary guidance to the formal consensus recommendations. UAL, ultrasound-assisted liposuction.

#### Postmarket Clinical Follow-up Survey Findings

A PMCF survey was conducted to provide descriptive context regarding early real-world use of the AYON UAL platform and was not used to develop or influence the Delphi consensus recommendations. A total of 213 PMCF surveys representing unique AYON UAL procedures performed across 19 clinical sites during early commercialization were analyzed to characterize early real-world use patterns. Because procedures could incorporate more than one technique classification, category totals exceed the number of reported cases. Most procedures were reported as standard UAL (156/213; 73.2%), with fewer cases categorized as high-definition UAL alone (25/213; 11.7%) or standard UAL combined with high-definition UAL techniques (40/213; 18.8%). Consequently, the summed counts exceed the total number of reported cases.

The system was reported to operate as intended for the planned procedure in 200/213 (93.9%) procedures. System performance issues were infrequent (13/213; 6.1%) and included challenges with emulsification in fibrous tissue (3/213; 1.4%), system malfunction (4/213; 1.9%), and other isolated concerns (6/213; 2.8%), including foot pedal problems (*n* = 2), prime button discrepancies (*n* = 1), a request for additional probe and cannula variety (*n* = 1), and issues not directly related to AYON UAL function (Renuvion screen issues, *n* = 2).

A total of 232 probe uses were reported across 213 procedures (Table 3). The 3.7 mm × 33 cm (5-ring) probe was used most frequently (63.8%), followed by the 3.7 mm × 26 cm (5-ring) probe (21.6%). Smaller-diameter probes were used less frequently. Multiple probes could be used during a single procedure; therefore, percentages exceed 100%.

**Table 3.** Postmarket Clinical Follow-Up Survey—Probe Utilization

Probe type	*n*/*N*	%^a^
2.2 mm × 18 cm—3 rings	14/213	6.6
2.9 mm × 18 cm—5 rings	17/213	8.0
2.9 mm × 26 cm—5 rings	19/213	8.9
3.7 mm × 26 cm—5 rings	46/213	21.6
3.7 mm × 33 cm—5 rings	136/213	63.8

^a^ *n* = number of procedures in which the probe was used. *N* = total procedures evaluated. Percentages exceed 100% because multiple probes could be used during a single procedure.

A total of 474 treatment areas were reported across 213 procedures (Table 4), reflecting treatment of multiple anatomic regions during a single procedure. The abdomen and pubis (66.2%), hips/flanks (49.3%), and back (40.8%) were the most frequently treated regions, followed by the legs, breast/axilla, and arms. Multiple treatment areas could be reported for each procedure; therefore, percentages exceed 100%.

**Table 4.** Postmarket Clinical Follow-Up Survey—Body Areas Treated

Body area	*n*/*N*	%^a^
Abdomen and pubis	141/213	66.2
Arms	33/213	15.5
Back	87/213	40.8
Buttocks	16/213	7.5
Breast and axilla	37/213	17.4
Face	3/213	1.4
Hips/flanks	105/213	49.3
Leg (thigh, knee, calf, ankle)	42/213	19.7
Neck	10/213	4.7

^a^ *n* = number of procedures in which the anatomical area was treated. *N* = total procedures evaluated. Percentages exceed 100% because multiple anatomical areas could be treated during a single procedure.

No concomitant procedures were reported in 12.7% of cases. Renuvion was the most frequently used adjunctive technology (48.4%), whereas other energy-based technologies were uncommon. Common concomitant surgical procedures included abdominoplasty (38.0%), fat transfer (28.6%), and mastopexy and breast augmentation (both 22.5%), with all other procedures reported less frequently (Table 5). Additional concomitant procedures reflected the diversity of aesthetic and reconstructive practice and included breast, facial, body contouring, and reconstructive procedures performed infrequently across individual cases. Because multiple concomitant procedures could be performed during a single case, percentages exceed 100%.

**Table 5.** Postmarket Clinical Follow-Up Survey—Concomitant Procedures

Concomitant procedure	*n*/*N*	%^a^
No additional procedures		
No other procedures were performed	27/213	12.7
Adjunctive energy-based technologies		
Renuvion (Apyx Medical, Clearwater, FL)	103/213	48.4
Ignite (InMode, Irvine, CA)	7/213	3.3
Morpheus (InMode, Irvine, CA)	3/213	1.4
Genius (Cynosure Lutronic, Westford, MA)	1/213	0.5
BodyTite/FaceTite (InMode, Irvine, CA)	0/213	0.0
Lumiia (Lumiia, Buenos Aires, Argentina)	0/213	0.0
Body-contouring procedures		
Abdominoplasty	81/213	38.0
Fat transfer	61/213	28.6
Brachioplasty	10/213	4.7
Thighplasty	5/213	2.3
Breast procedures		
Mastopexy	48/213	22.5
Breast augmentation	48/213	22.5
Breast reduction	11/213	5.2
Implant exchange	10/213	4.7
Gynecomastia surgery	2/213	0.9
Face and neck procedures		
Necklift	4/213	1.9
Facelift	3/213	1.4
Blepharoplasty	2/213	0.9
Rhinoplasty	0/213	0.0
Skin resurfacing	4/213	1.9
Nonsurgical cosmetic procedures (toxins/fillers)	2/213	0.9

^a^ *n* = number of procedures in which the concomitant procedure was performed. *N* = total procedures evaluated. Percentages exceed 100% because multiple concomitant procedures could be performed during a single procedure.

Surgeons reported that no adverse events occurred during the procedure in 212 cases (212/213; 99.5%). A single intraoperative adverse event was reported (1/213; 0.5%), consisting of bleeding at the left inner knee that did not require transfusion.

## DISCUSSION

This modified Delphi consensus provides an initial framework for the clinical use of the AYON UAL platform. In the absence of published device-specific procedural guidance, these recommendations synthesize expert practice patterns to support procedural standardization, clinical education, and future research while recognizing that prospective comparative studies remain necessary.^11,17^ Although UAL is well established for adipose tissue fragmentation, the AYON UAL platform incorporates integrated workflow features and real-time feedback mechanisms that warrant device-specific guidance.^2,3,14^

The consensus findings reinforce that the core principles governing safe and effective UAL remain applicable with contemporary platforms. Adequate infiltration, tactile feedback during ultrasound application, and adaptation of power and probe selection to tissue characteristics remain the primary determinants of treatment strategy, consistent with foundational descriptions of UAL.^18^ Accordingly, AYON UAL represents an evolution of established UAL practice incorporating system-level refinements to support procedural efficiency and consistent energy delivery. The integrated architecture of the AYON platform facilitates procedural organization, whereas standardized workflows and staff familiarity were considered important for procedural efficiency, safety, and adoption.

Adjunctive EBDs were frequently incorporated following liposuction to address skin laxity and contour refinement. Because the AYON platform accommodates helium plasma RF as an integrated adjunctive technology, helium plasma RF was the most frequently discussed adjunctive modality during the consensus process. However, the Delphi methodology was not designed to compare helium plasma RF with other available EBDs or to evaluate whether platform integration influenced surgeon selection of adjunctive technologies. Rather, the consensus focused on technical considerations for incorporating adjunctive energy-based treatments into UAL workflows. These observations align with broader trends toward multimodal body contouring and emerging clinical evidence evaluating combined UAL and helium plasma RF approaches.^19^ In a recent retrospective study, the addition of helium plasma RF following third-generation UAL during abdominoplasty was not associated with an increase in reported adverse events compared with UAL treatment of the entire flap alone.^19^ Although these findings are encouraging, prospective comparative studies remain warranted.

The PMCF survey complements the consensus findings by providing descriptive insight into early real-world use patterns. Applications included standard liposuction, high-definition liposculpture, fat harvest, and integration of adjunctive energy-based technologies, reflecting the broad procedural applications described by the expert panel.

The PMCF safety observations were consistent with the panel's emphasis on structured procedural safeguards, with infrequent intraoperative adverse events and device performance issues. However, these findings should be interpreted cautiously given the descriptive design, the absence of longitudinal follow-up, and the lack of a comparator group.

This consensus paper has limitations inherent to its design. As a modified Delphi process, the findings reflect expert opinion rather than outcomes derived from controlled clinical trials, and the panel size may not capture the full range of practice patterns across clinical settings. These recommendations should therefore be viewed as expert consensus guidance rather than evidence-based clinical practice guidelines. Additionally, all panelists served as consultants to the study sponsor and had previous experience with the AYON UAL platform, representing a potential source of bias despite the use of a structured, predefined consensus methodology. Consensus recommendations were also based on self-reported experience and technique preferences, which may vary according to training background, procedural volume, and practice environment. The accompanying PMCF survey captured procedural and immediate perioperative observations only and did not include longitudinal follow-up; therefore, delayed adverse events or longer-term outcomes may be underrepresented.

Prospective clinical studies with standardized follow-up will be important to further characterize clinical outcomes and safety across broader patient populations and practice settings. Until higher-level clinical evidence becomes available, these consensus recommendations provide a practical framework to support clinical adoption, procedural standardization, and future evidence generation.

## CONCLUSIONS

AYON UAL is an integrated UAL platform designed to support contemporary body-contouring workflows by consolidating infiltration, ultrasonic fragmentation, and aspiration within a single system that also accommodates helium plasma RF technology. Using a structured modified Delphi methodology, this expert panel developed device-specific consensus recommendations for key aspects of AYON UAL use, including procedural workflow, power and probe selection, anatomic considerations, and procedural safety. Early PMCF survey findings provide descriptive context regarding initial real-world use. Together, these findings provide a practical framework to support clinical adoption, procedural standardization, education, and future clinical research.
